# Salt-tolerance screening in *Limonium sinuatum* varieties with different flower colors

**DOI:** 10.1038/s41598-021-93974-3

**Published:** 2021-07-15

**Authors:** Xiaojing Xu, Yingli Zhou, Ping Mi, Baoshan Wang, Fang Yuan

**Affiliations:** Shandong Provincial Key Laboratory of Plant Stress, College of Life Sciences, Shandong Normal University, Ji’nan, Shandong People’s Republic of China

**Keywords:** Molecular biology, Plant sciences

## Abstract

*Limonium sinuatum*, a member of Plumbaginaceae commonly known as sea lavender, is widely used as dried flower. Five *L. sinuatum* varieties with different flower colors (*White*, *Blue*, *Pink*, *Yellow*, and *Purple*) are found in saline regions and are widely cultivated in gardens. In the current study, we evaluated the salt tolerance of these varieties under 250 mmol/L NaCl (salt-tolerance threshold) treatment to identify the optimal variety suitable for planting in saline lands. After the measurement of the fresh weight (FW), dry weight (DW), contents of Na^+^, K^+^, Ca^2+^, Cl^−^, malondialdehyde (MDA), proline, soluble sugars, hydrogen peroxide (H_2_O_2_), relative water content, chlorophyll contents, net photosynthetic rate, and osmotic potential of whole plants, the salt-tolerance ability from strongest to weakest is identified as *Pink*, *Yellow*, *Purple*, *White*, and *Blue*. Photosynthetic rate was the most reliable and positive indicator of salt tolerance. The density of salt glands showed the greatest increase in *Pink* under NaCl treatment, indicating that *Pink* adapts to high-salt levels by enhancing salt gland formation. These results provide a theoretical basis for the large-scale planting of *L. sinuatum* in saline soils in the future.

## Introduction

*Limonium sinuatum* L., a native Mediterranean plant, is widely distributed in Northern Africa, western Asia, and Europe^1^. *L. sinuatum*, a member of the Plumbaginaceae family, is a typical recretohalophyte that can grow in saline^2^ or drought environments due to the presence of salt glands in the epidermis and the surrounding thick cuticle to reduce the water evaporation^3^. Different *L. sinuatum* varieties are usually identified based on flower color. The flower petals are white, while the calyxes can be different colored (e.g., white, blue, pink, yellow, and purple)^4^. The calyxes remain long after the petals have disappeared, making the flowers attractive for long periods of time and excellent for use as fresh cut flowers or in dried arrangements^5^. The flower stems are approximately 40–50 cm tall, each flower stem has 2–3 branches with 5–6 flowers clustered together, and the diameter of each flower is ~ 0.5 cm^6^. *L. sinuatum* is commonly referred to as statice, sea lavender, sea notchleaf^7^, or wavyleaf sea lavender when used for gardening or floral arrangements^7^. Moreover, the entire plant is used as a traditional Chinese medicine for hemostasis^8^. In China, *L. sinuatum* is widely distributed along the coast of the Yellow Sea and the Bohai Sea^9^.

Soil salinization is a major environmental factor affecting plant growth and development^10^. Plant salt tolerance is a highly complex trait involving many factors, such as tissue and organ structure and physiological and biochemical reactions^11^. Salt stress alters the contents of soluble sugars, ions, and proline in plants^12^, and it affects the synthesis of plant soluble substances^13^, leading to changes in salt tolerance^14^.

Most crops are non-halophytes, and their growth is inhibited in saline soil^15^. By contrast, halophytes, including euhalophytes, recretohalophytes, and psudohalophytes^16^, can grow and complete their lifecycles in the presence of ≥ 200 mmol/L NaCl^17^. *L. sinuatum* is a recretohalophyte with salt glands for excreting excess Na^+^ out of the plants to avoid salt stress. To date, 67 species with salt glands have been reported, belonging to 13 families^18^. Among Plumbaginaceae family members, many studies have been carried out on *L. bicolor*, including salt secretion measurements^19^, analysis of salt gland differentiation^20^, and transcriptomic analysis during leaf development and in response to NaCl treatment^16^. However, although *L. sinuatum* also belongs to *Limonium*, few studies of the salt resistance in different varieties of this plant have been reported.

Generally, *L. sinuatum* has greater potential than *L. bicolor* for use as a horticultural crop in saline soils^20^, because the growth cycle of *L. sinuatum* is short (do not need vernalization for flowering) and the flower color is various, and it is more suitable for gardening^21^. Given that *Limonium* species are considered to be pioneer plants for transforming saline soils^22^, it is important to explore the use of these plants to maximize the utilization of saline lands to increase the economic and ecological value of these environments. Here, we selected five common garden varieties of *L. bicolor* (named based on flower color), including *White*, *Blue*, *Pink*, *Yellow*, and *Purple*, and determined their salt-tolerance thresholds. We evaluated the salt tolerance of these varieties by comparing fresh weight, dry weight, ion, proline, soluble sugars, and chlorophyll content, net photosynthetic rate, and osmotic potential to identify the varieties with the strongest salt tolerance. Based on these salt-tolerance indicators, we identified the most suitable variety for planting in saline soil.

## Materials and methods

### Plant materials and growth conditions

**Figure 1:**
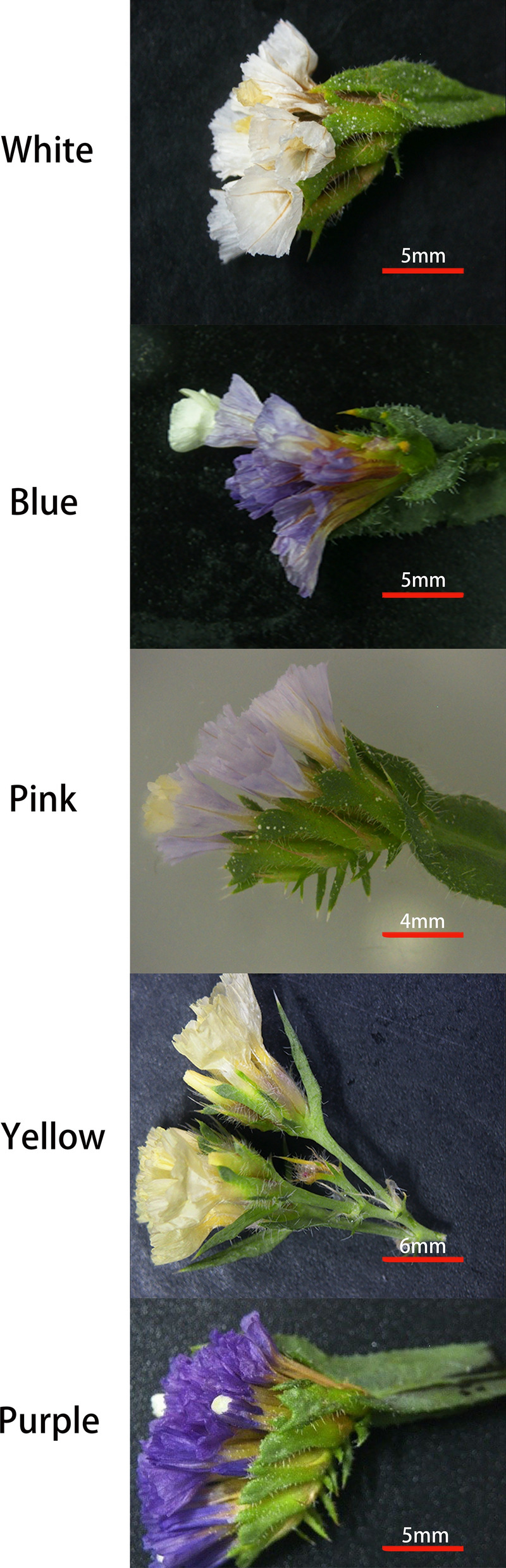
Flower color in different varieties of *Limonium sinuatum* after 6 months of growth. The photographs show flowers from the *White*, *Blue*, *Pink*, *Yellow*, and *Purple* varieties. Different colors indicates the calyx’s colors.

The seeds of five *Limonium sinuatum* varieties with different flower colors (*White*, *Blue*, *Pink*, *Yellow*, and *Purple*) were purchased from Lanxiang Horticulture Seedling Co., Ltd. (China). This study complies with local and national regulations. The author Baoshan Wang had formally identified *L. sinuatum*, and the seeds harvesting process is in full compliance with relevant government guidelines. Unfortunately, we were unable to find a voucher specimen of *L. sinuatum* stored in any publicly available herbarium. The dried seeds were stored in a refrigerator at < 4 °C. The seeds were sterilized in 6% NaClO for 15 min, washed with sterile distilled water, and sown in nutrient soil (soil: vermiculite: perlite, 3: 1: 1). The plants were grown in a growth chamber at 28 °C/23 °C (day/night) under 600 μmol/m^2^/s full-spectrum light (15 h photoperiod) and 60% relative humidity. In order to make accurate comparisons among the five varieties, all plants were cultured for six months under the above conditions for flowering (Fig. 1). Flower color can be used to distinguish among different varieties of *L. sinuatum*, including *White*, *Blue*, *Pink*, *Yellow*, and *Purple*.

### Measurement of salt-tolerance threshold

To determine the salt-tolerance threshold, two-week-old seedlings with two expanded leaves were treated with different concentrations of NaCl (0, 100, 200, 300 and 400 mmol/L). After two weeks of treatment, the aerial parts of the plants were collected and used to measure fresh weight (FW) and dry weight (DW) according to Huang et al.^23^. Five replicates were performed per variety, and the means among different varieties under each NaCl treatment were used to calculate the salt-tolerance threshold. In detail, a fit regression curve was established with different NaCl concentrations *vs.* FW or DW. The NaCl concentration at which the plants showed 50% FW or DW^24^ compared to the non-NaCl treatment group was considered to be the salt-tolerance threshold.

After the determination of salt-tolerance threshold, two-week-old seedlings of the five varieties with two expanded leaves were then re-treated with 0 mmol/L and 250 mmol/L NaCl (considered to be the salt-tolerance threshold in the following experiments) for two weeks and used to measure the physiological indicators.

### Determination of physiological indicators

#### Determination of FW, DW and relative water content of leaf.

After cleaning the leaves with 10 mM calcium chloride solution^25^ followed by deionized water, FW of the leaves was measured immediately and DW was obtained following incubation at 105 °C for 15 min and drying to constant weight at 70 °C for 2 days^23^. Five replicates were performed per variety and treatment. The reduction rate was calculated as (FW under control condition—FW under saline condition)/FW under control condition × 100%. The same method was processed in calculating the reduction rate of DW. The relative water content is calculated as (FW-DW)/FW × 100%^26^.

#### Determination of sodium ion (Na^+^), potassium ion (K^+^), calcium ion (Ca^2+^), and chloride ion (Cl^−^) contents.

The ion contents in the samples were measured according to Higinbotham^27^. In brief, leaf tissue (0.5 g FW) was collected from plants under both 0 and 250 mmol/L NaCl treatment for all five varieties. The tissues were ashed, dissolved in HNO_3_, and the contents of Na^+^, K^+^, and Ca^2+^ measured using a Flame photometer (FP6440, Yuanxi, Shanghai, China). Cl^−^ content was measured by ion chromatography according to Wang^28^. Briefly, after boiling for 30 min and filtering through a 0.22 μm filter membrane, the ion solution was injected into an ion chromatograph (ICS-90A, ThermoFisher, Massachusetts, USA) to measure Cl^−^ contents. The ion concentration is shown as mmol/g FW. Five replicates were performed for each variety and treatment. Given that Na^+^ content increased under NaCl treatment, the increase rate was calculated as (Na^+^ content under saline condition—Na^+^ content of control)/Na^+^ content of control × 100%. The same calculation method was applied in the increase rate of Cl^−^.

#### Determination of proline content, osmotic potential, malondialdehyde (MDA), hydrogen peroxide (H_2_O_2_), soluble sugars, and chlorophyll content, and photosynthetic rate.

Proline content was determined in accordance with Demiral^29^. The plant tissue was ground, and ground tissue (0.5 g FW) was added to 10 mL of 5% acetic acid and 40 mL of distilled water. After filtering, the filtrate (8 mL) was mixed with 0.8 g zeolite with shaking for 5 min and centrifuged for 10 min (1500 g). The supernatant (3 mL) was combined with glacial acetic acid (3 mL) and ninhydrin reagent (3 mL) and boiled for 1 h. Benzene (3 mL) was used for static layering, and the upper colored liquid was collected and used to measure optical density at 515 nm. The proline content was calculated from a standard curve based on the optical density. Five replicates were performed for each variety and treatment.

The osmotic potential was measured as described by Tomlinson^30^. Fresh leaf tissue (0.5 g) was cut into small pieces, frozen in liquid nitrogen, and placed into a syringe to squeeze out and collect the cell sap. A freezing point osmometer (SMC 30C-1, Tianhe, Tianjin, China) was used to measure the osmotic potential of the plant cell sap. The formula used to calculate osmotic potential is − iCRT (R = 0.0083143 L Mpa Mol^−1^ K^−1^, T = 298 K). Five replicates were performed for each variety and treatment.

The MDA content was determined as reported in Hong^31^. Leaf tissue (0.5 g) was collected and homogenized in 5 mL 0.1% TCA. The homogenate was transferred to the test tube, combined with 5 mL 0.5% thiobarbituric acid solution, and boiled for 10 min. The sample was centrifuged at 1500 g for 15 min, and the optical density of the supernatant was measured at 532 nm and 600 nm. MDA content (mmol/g FW) = ΔAN/155 W, ΔA is the difference between A _532_ and A _600_; N is the total volume of the supernatant; 155 is the absorption coefficient of 1 mmol reaction product at 532 nm; W is the fresh weight of the plant material (g). Five replicates were performed for each variety and treatment.

The content of H_2_O_2_ was determined as described by Vergara^32^. In brief, fresh leaves (0.3 g) was grinded in 5 mL precooled acetone before centrifuged at 500 g for 8 min. Afterward the supernatant (1 mL) was mixed with ammonia (0.2 mL) and 20% TiCl_4_ (0.1 mL) for 2 min, the precipitate was washed with acetone for 3–5 times and dissolved in 2 M H_2_SO_4_ (5 mL) after centrifuged at 600 g for 7 min. Then the content of H_2_O_2_ was measured at 415 nm and calculated as H_2_O_2_ content (μmol/g FW) = CV_T_/FWV_1_, C is the concentration of H_2_O_2_ in the sample checked on the standard curve (μmol), V_T_ was the total volume of sample extract (mL), V_1_ was the volume of sample extract (mL), and FW was the fresh weight of plant tissue (g). Five replicates were performed for each variety and treatment.

Soluble sugars were measured following the protocol of Prado^33^. Fresh leaf tissue (0.3 g) was dissolved in 10 mL of double distilled H_2_O (ddH_2_O) in a boiling water bath for 50 min, filtered, and brought to a volume of 25 mL. Afterward 0.5 mL of extract solution was combined with 1.5 mL distilled water, 0.5 mL ethyl anthrone acetate, and 5 mL concentrated sulfuric acid, shaken thoroughly, boiled in water bath for 1 min, and cooled. The optical density of the solution was measured at 630 nm. The soluble sugars content was calculated from a standard curve. Five replicates were performed for each variety and treatment.

Chlorophyll levels were determined referring to Maxwell^34^. Leaf tissue (0.3 g) was combined with 5 mL dimethyl sulfoxide in 5 mL 80% acetone and incubated in a 65°C water bath at 24 h (protected from the light) to fully decolorize. Afterward bring to 25 mL after filtration and the solution was used to measure the optical density at 663 nm, 645 nm, and 470 nm. Chlorophyll content (mg g^−1^ or mg dm^−2^) = CV/1000A, C is chlorophyll concentration (mg L^−1^ or mg dm^−2^); V is the total volume of extract solution (mL); A is fresh weight of the sample (g) or sampling area (dm^−2^). The pigment concentration (mg/L) was calculated as C_*a*_ = 12.7A_663_ − 2.69A_645_; C_*b*_ = 22.9A_645_ − 4.68A_663_; C_total_ = 20.0A_645_ + 8.02A_663_; C_XC_ = (1000A_470_ − 3.27C_*a*_ − 104C_*b*_)/229; C_*a*_, C_*b*_ are the concentrations of chlorophyll *a* and *b*, C_total_ is the concentration of total chlorophyll; C_XC_ is the total concentration of carotenoids. Five replicates were performed for each variety and treatment.

The photosynthetic rate was measured on the basis of Wang^35^. In this experiment, a photosynthetic instrument (LI-6400XT, LI-COR, Nebraska, USA) was used to measure the photosynthetic parameters of leaves. The photosynthetic effective quantum density, U_PAR_ (μmol m^−1^ s^−1^), μ is 4.55^36^, was measured at a temperature of 23°C, and the leaf area of each cultivar was 1 cm^2^. Five replicates were performed for each variety and treatment.

### Determination of salt gland density in different varieties

The density of salt glands was measured according to Yuan^37^. The leaves were fixed in a mixture of ethanol and acetic acid (3:1; v/v), rinsed with 70% ethanol to decolorize, and cleared in Hoyer’s solution. Afterward cleaned leaves were fixed on a glass slide for DIC microscopy (ECLIPSE 80i, Nikon, Tokyo, Japan). The salt gland density was calculated according to Ding^38^ and expressed as number per mm^2^. Five replicates were performed for each variety and treatment.

## Data analysis

Statistical analysis and correlation were performed using SPSS 13.0 software (SPSS Software Inc., USA). The results were subjected to a one-way analysis of variance (ANOVA), and Duncan’s test was used to determine significant differences between the means (*P* = 0.05). In the figures, the error bars represent the means ± standard deviations (n = 5) and different letters indicate significant differences at *P* = 0.05. Correlation is processed at *P* = 0.05 and 0.01 using Pearson correlation analysis. The figures were generated using SigmaPlot 12.5 (Systat Software, Chicago, IL, USA).

## Results

### Identification of the salt-tolerance threshold

**Figure 2:**
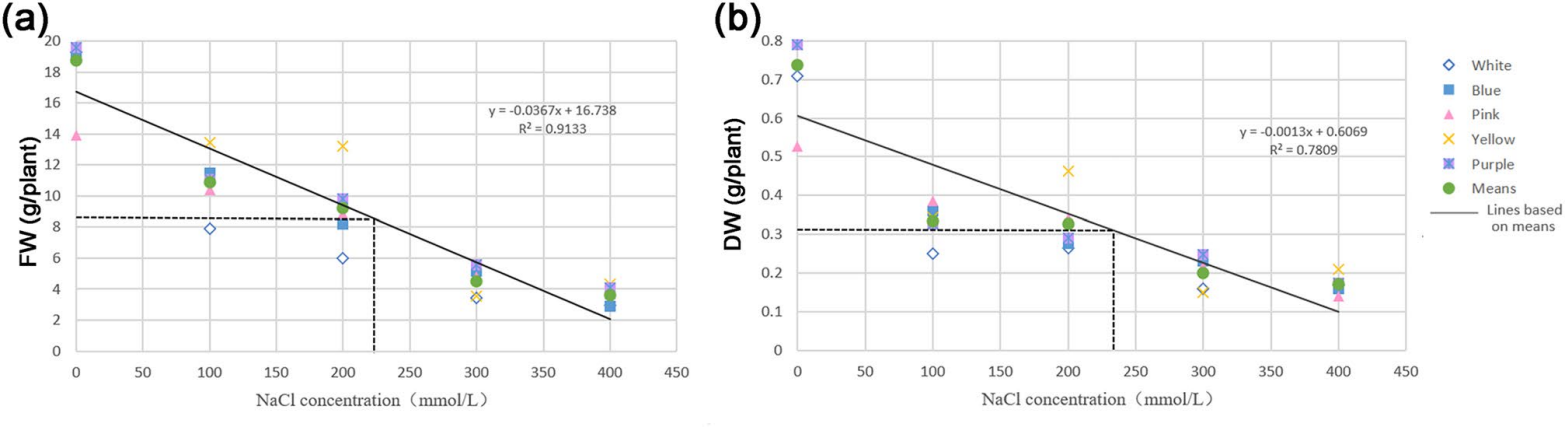
Salt-tolerance thresholds of five varieties of *Limonium sinuatum*. A 50% reduction in biomass compared to the control was used as the standard to determine the salt-tolerance threshold. (**a**) Changes in fresh weight (FW) of the leaves of five *L. sinuatum* varieties under different salt treatments. (**b**) Changes in dry weight (DW) of the leaves of five *L. sinuatum* varieties under different salt treatments.

Plant biomass is an important measure of salt tolerance. Figure 2 shows the biomass of the aerial parts of plants under a gradient of different NaCl concentrations (0, 100, 200, 300, and 400 mmol/L) after 2 weeks of treatment. FW and DW were measured in the five varieties of *L. sinuatum* seedlings and constructed a regression curve based on the means of five data under different treatments as independent variables. Most studies use the salt concentration at which plant growth or biomass decreases by 50% of the control value as the salt-tolerance threshold^39^. Here, when the FW and DW of the upper parts of the seedlings were reduced by 50% of the non-NaCl treatment value, different varieties showed different salt-tolerance thresholds. The highest threshold was obtained for *Pink* (250 mmol/L) (Supplementary Fig. 1), suggesting that *Pink* is the most salt-tolerant variety. To identify the optimal salt concentration for further experiments, we calculated the average salt-tolerance threshold, i.e., 228 mmol/L for FW and 233 mmol/L for DW (Fig. 2). Therefore, a salt-tolerance threshold of 250 mmol/L was utilized in subsequent experiments.

### Pink shows the best growth under 250 mmol/L NaCl treatment

**Figure 3:**
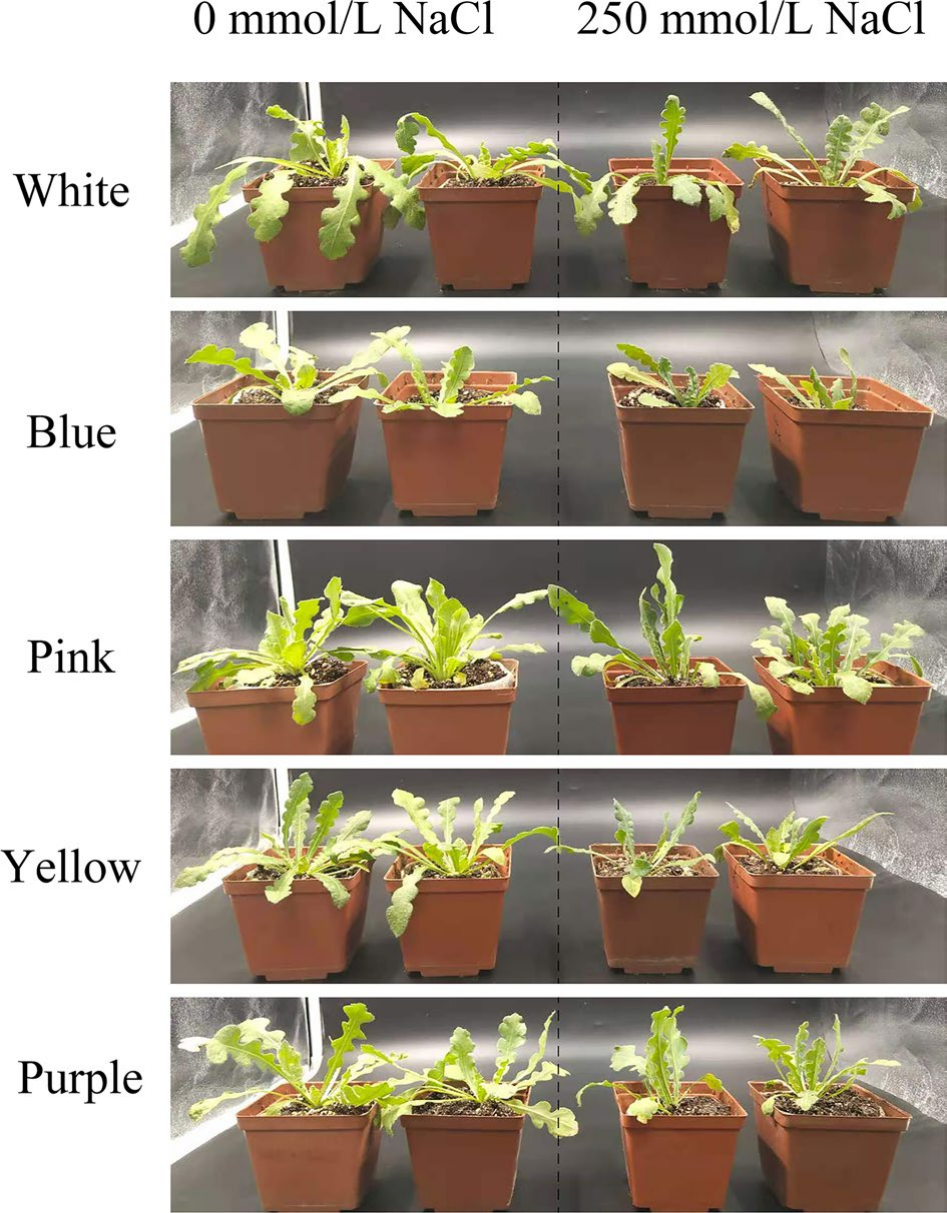
Growth of five varieties of *Limonium sinuatum* seedlings after two weeks of salt treatment.

**Figure 4:**
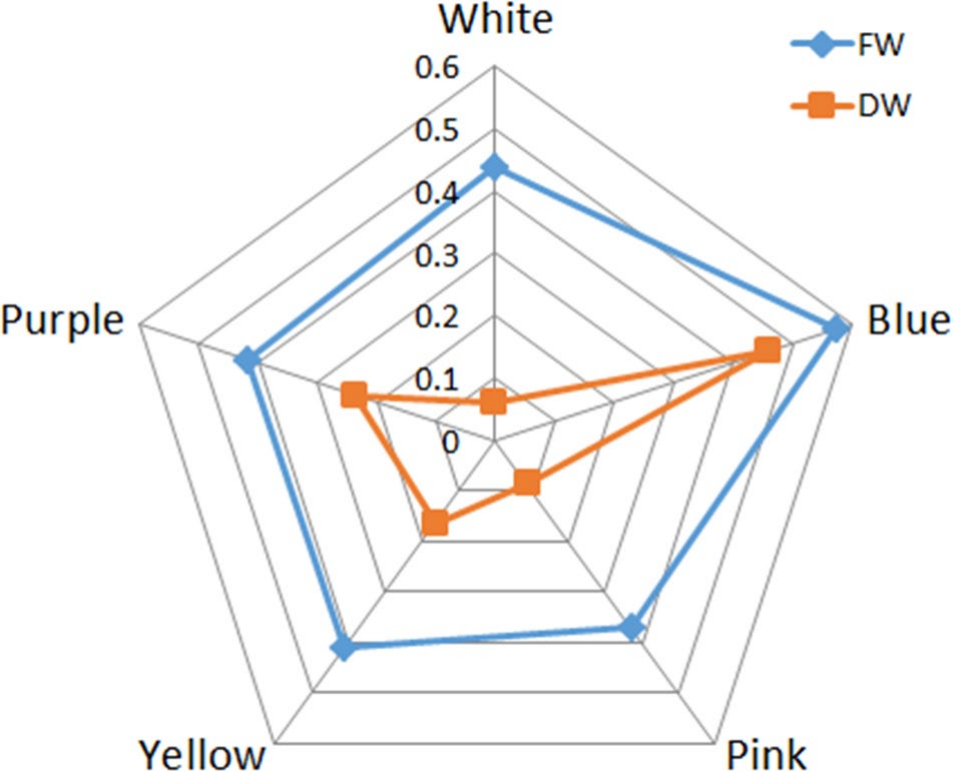
Effect of NaCl stress on the reduction rate of fresh weight (FW) and dry weight (DW) of the leaves of five *Limonium sinuatum* varieties. The reduction rate was calculated as (FW or DW under control condition − FW or DW under saline condition)/FW or DW under control condition × 100%.

All varieties showed inhibited growth under 250 mmol/L NaCl treatment (Fig. 3), but the changes in FW and DW showed no significant trends among varieties (Supplementary Fig. 2). In order to make effective comparison among different varieties, FW and DW reduction rate are calculated to compare the changes between control and saline condition (Fig. 4). *Pink* showed the least FW reduction, followed by *Yellow*, *Purple, White* and *Blue*, while *White* has the least DW reduction, afterward *Pink*, *Yellow*, *Purple* and *Blue*. Based on the reduction rate of FW and DW, *Pink* is considered the most salt tolerance variety, followed by *Yellow*, *Purple*, *White*, and *Blue*. Biomass can be used as a measure of plant growth, and various physiological processes could be responsible for the ability of *Pink* to maintain growth in the presence of salt. Therefore, we measured the physiological indicators of the different varieties under NaCl treatment in order to reveal the underlying salt-tolerance mechanisms.

### Effect of NaCl treatment on different physiological indicators in five varieties

**Figure 5:**
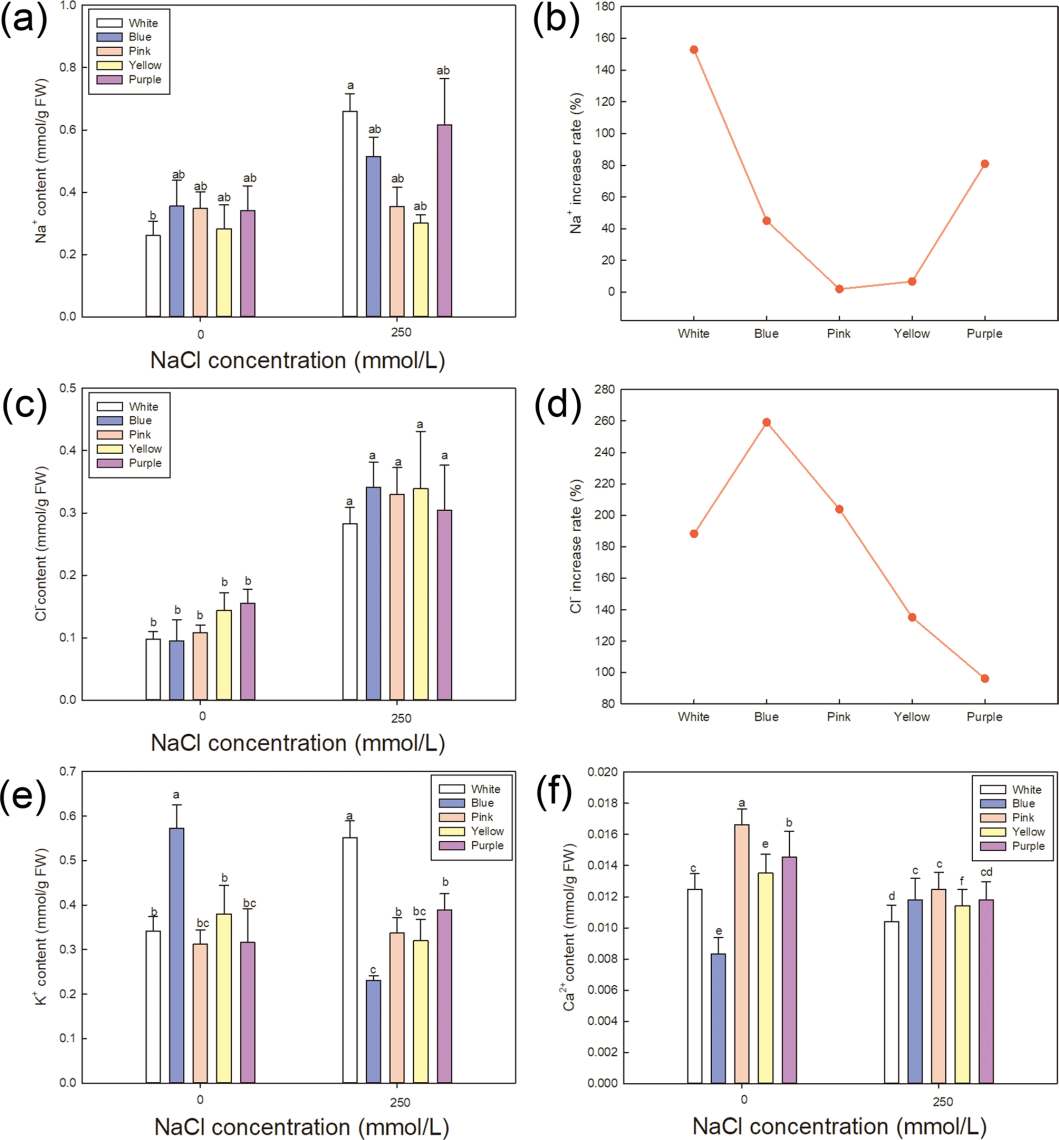
Effect of NaCl treatment on Na^+^, K^+^, Ca^2^^+^, and Cl^−^ contents. Contents of Na^+^ (**a**), Cl^−^ (**c**), K^+^ (**e**) and Ca^2^^+^ (**f**) under control and NaCl treatment in different varieties. The data are means ± SD of five replicates. Different letters indicate significant differences between two groups at *P* = 0.05 using Duncan’s test with SPSS. (**b**, **d**) The increase rate of Na^+^ and Cl^−^ under NaCl treatment in five varieties, which was calculated as (ion contents under NaCl treatment—ion contents under control)/ion contents under control × 100%.

**Figure 6:**
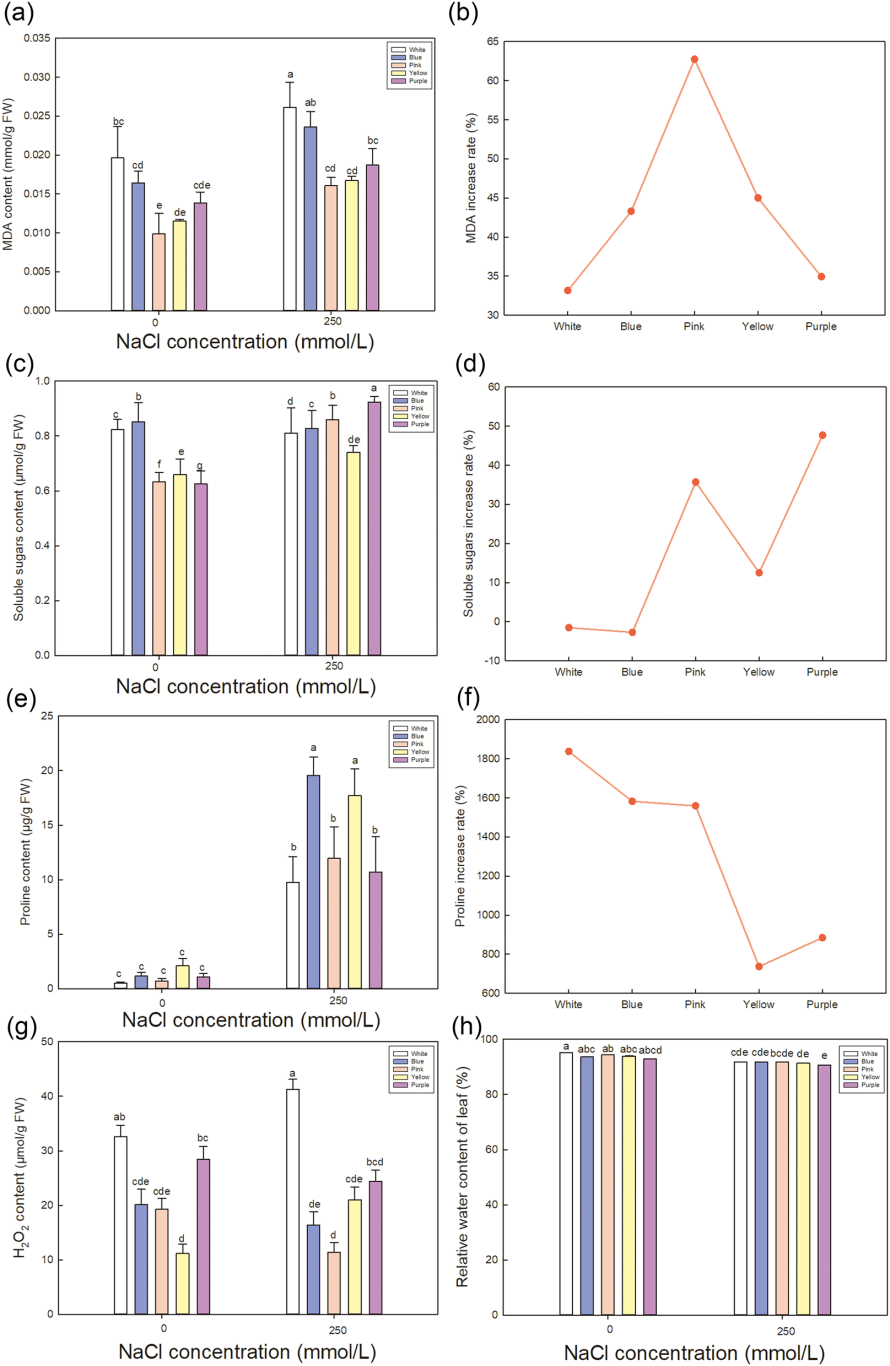
Effect of NaCl stress on MDA, soluble sugars, proline contents, H_2_O_2_ and relative water content in the leaves of five varieties of *Limonium sinuatum*. Contents of MDA (**a**), soluble sugars (**c**), proline (**e**), H_2_O_2_ (**g**) and relative water content (**h**) of leaves under control and NaCl treatment in different varieties. The data are means ± SD of five replicates. Different letters indicate significant differences between two groups at *P* = 0.05 using Duncan’s test with SPSS. The increase rate of MDA (**b**), soluble sugars (**d**) and proline (**f**) are calculated using (value under NaCl treatment—value under control)/value under control × 100%.

**Figure 7:**
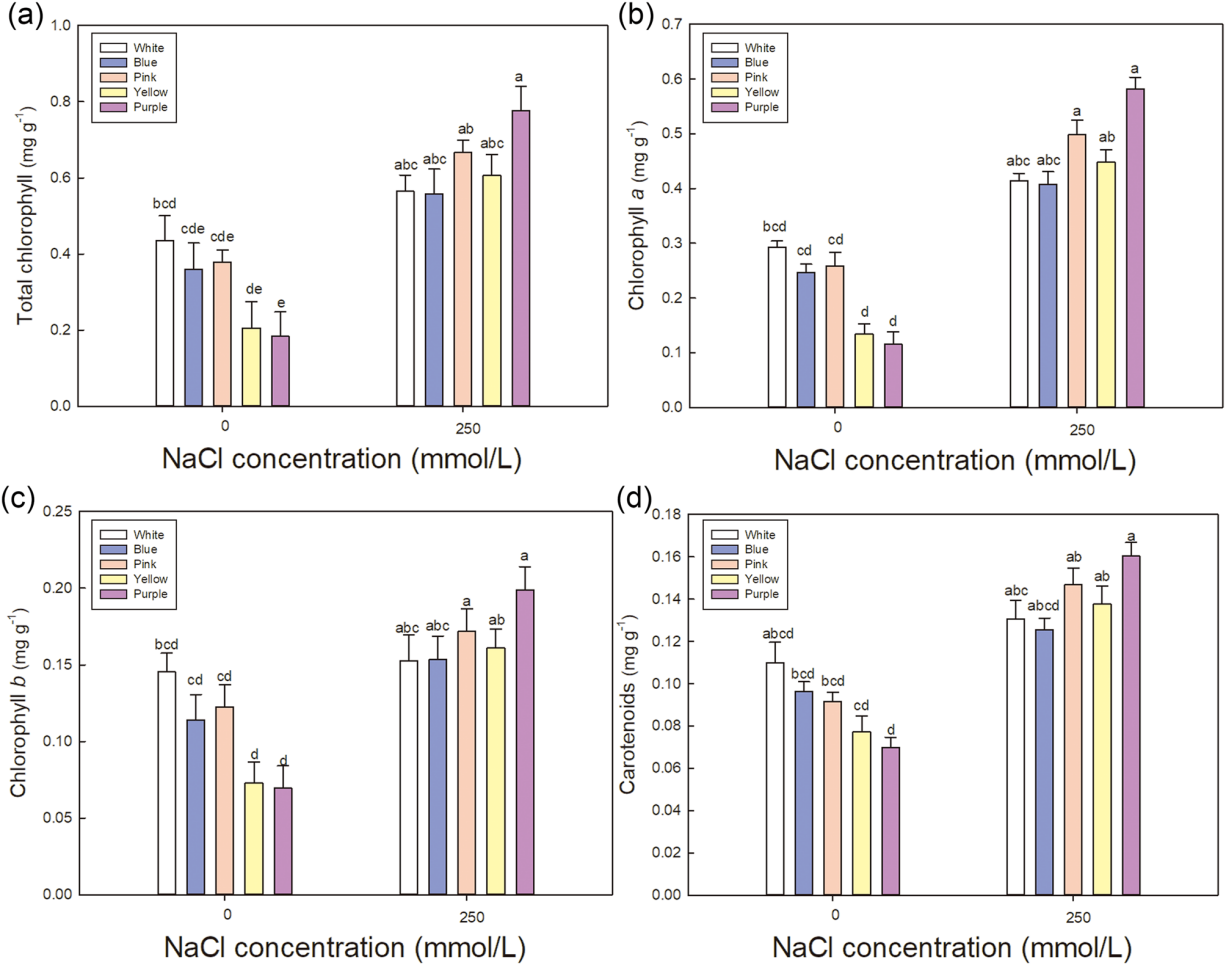
Effect of NaCl treatment on chlorophyll contents in the leaves of five varieties of *Limonium sinuatum*. (**a**–**d**) Total chlorophyll, chlorophyll *a*, chlorophyll *b*, and carotenoid contents under control and NaCl treatment. The data are means ± SD of five replicates. Different letters indicate significant differences between two groups at *P* = 0.05 using Duncan’s test with SPSS.

**Figure 8:**
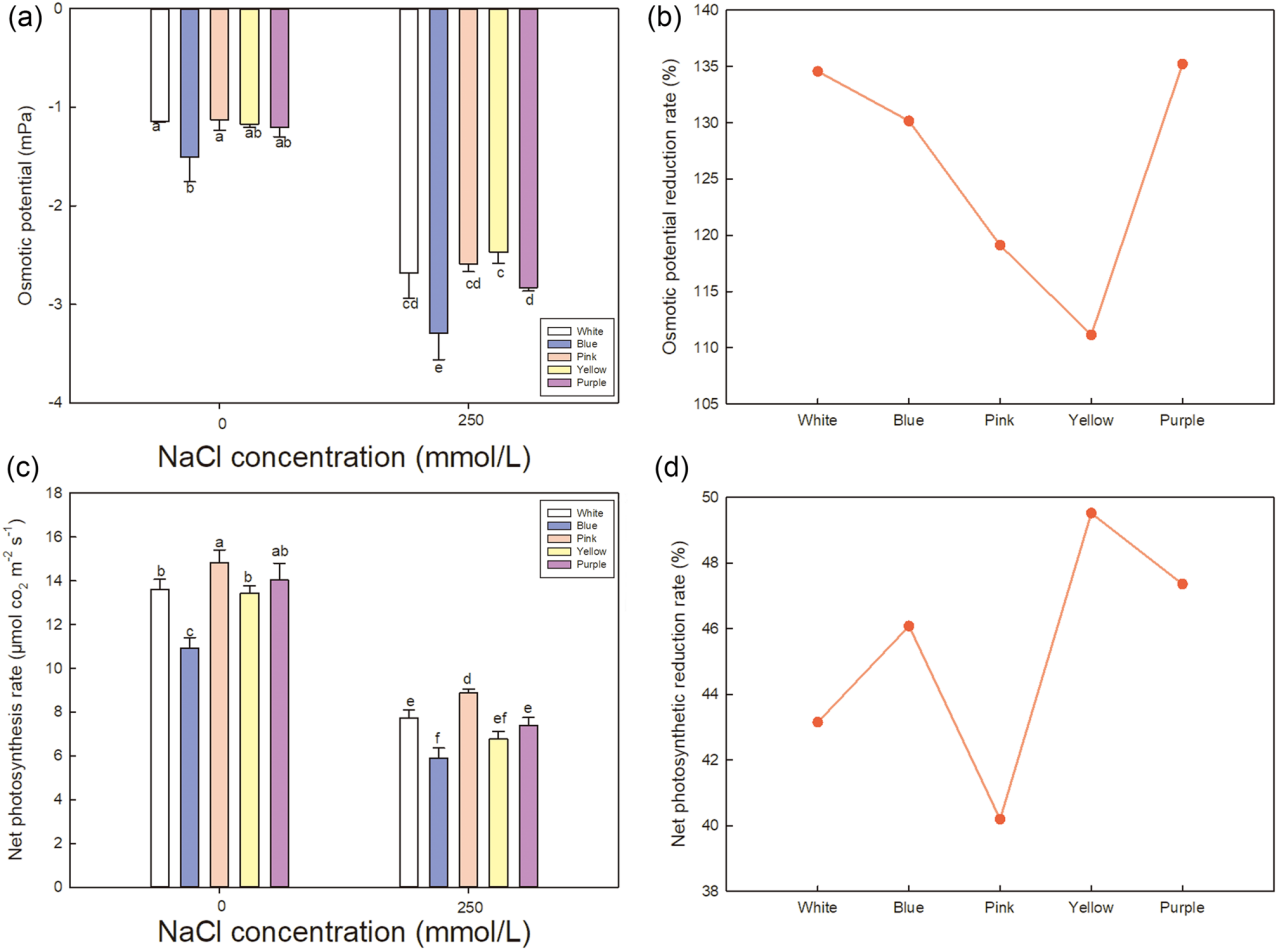
Effect of NaCl stress on the osmotic potential and net photosynthetic rate in the leaves of five varieties of *Limonium sinuatum*. (**a**, **c**) Osmotic potential and net photosynthetic rate under control and NaCl treatment. The data are means ± SD of five replicates. Different letters indicate significant differences between two groups at *P* = 0.05 using Duncan’s with SPSS. (**b**, **d**) The reduction rate of osmotic potential and net photosynthetic rate under NaCl treatment. The reduction rate was calculated as (the value under control—the value under NaCl treatment)/the value under control × 100%.

Comparisons of the Na^+^, K^+^, Ca^2+^, and Cl^−^ contents; MDA, soluble sugars, proline contents, H_2_O_2_ content and relative water content of leaf; chlorophyll contents; and osmotic potential and photosynthetic rate are shown in Figs. 5, 6, 7 and 8, respectively. Each variety showed significant changes under NaCl treatment.

Figure 5 shows a comparison of the relative Na^+^, K^+^, Ca^2+^ and Cl^−^ contents among varieties under 250 mmol/L NaCl treatment. Na^+^ and Cl^−^ content under NaCl treatment increased compared with the control in all varieties (Fig. 5a,c), while K^+^ and Ca^2^^+^ showed various trends in different varieties (Fig. 5e,f). Given that Na^+^ and Cl^−^ are considered the stress ion to protoplast and different varieties have various basal level under control, the increase rate of Na^+^ (Fig. 5b) and Cl^−^ (Fig. 5d) under NaCl treatment are calculated in each variety in order to make intuitive comparison among different varieties. *Pink* shows the least Na^+^ increase (1.87%) under saline condition, followed by *Yellow*, *Blue*, *Purple* and *White*. In the aspect of Cl^−^ increase, *Purple* (96.04%) has the least, afterward *Yellow*, *Pink*, *White* and *Blue*. Together with the increase rate of Na^+^ and Cl^−^, these results indicate that *Pink* accumulates less Na^+^ and Cl^−^ than the other varieties under high-salt conditions, which should lead to less injury than the other varieties.

Figure 6 shows a comparison of the relative MDA, soluble sugars, proline contents, H_2_O_2_ and relative water content of leaf among varieties. Though the MDA increase rate of *Pink* under saline condition (Fig. 6b) shows the most, the absolute value of MDA (Fig. 6a) is the least accumulation in *Pink*, indicating that *Pink* suffered the least amount of damage under salt treatment, as MDA can be used as a measure of the degree of damage under NaCl treatment^40^. High accumulation of MDA can be detected in *White* and *Blue* (Fig. 6a), which may explain the serious damage level.

To cope with the damage caused by NaCl treatment, cells usually accumulate organic osmotic regulating substance such as soluble sugars (Fig. 6c) and proline (Fig. 6e). High accumulation of soluble sugars is shown in *Pink* and *Purple*, and the increase rate (Fig. 6d) under saline treatment indicates the comparison between varieties. Highest increase rate is detected in *Purple*, followed by *Pink* and *Yellow*. Besides, high proline accumulation is shown in *Blue*, *Yellow* and *Pink* in descending order of actual value (Fig. 6e), while the increase rate has the opposite trend with the most in *White*, *Blue* and *Pink* (Fig. 6f). Proline reduces the osmotic potential in the cell^41^, allowing it to resist external osmotic stress, thereby improving plant survival in adverse environments^29^. Proline content depends on the catabolism of sugar^42^.Combined the absolute value and the increase rate, *Pink* is considered to accumulate a large amounts of soluble sugars and proline to improve the osmotic adjustment ability under salt treatment. The large accumulation of osmoregulation substances can effectively reduce the osmotic potential under NaCl treatment^43^. Figure 8 (a) shows that the osmotic potential decline markedly in all varieties, and *Yellow* and *Pink* have the most reduction rate (Fig. 8b).

Moreover, H_2_O_2_, as a kind of superoxide, can cause oxidative stress to plants under various stresses^44^. In Fig. 6g, under salt stress, the lowest H_2_O_2_ generation is detected in *Pink*, while higher in *White* and *Purple*, which indicates that *Pink* suffers the least oxidative stress under salt treatment and is more suitable for saline environment. In addition, relative water content is also measured (Fig. 6h) and no significant difference is detected among different varieties, indicating that all varieties of *L. sinuatum* can keep normal moisture condition to cope with physiological drought of NaCl^45^.

Plants always produce large amounts of pigments under saline environment to maintain normal photosynthetic efficiency. In addition, a positive correlation was detected between chlorophyll content and net photosynthetic rate^46^. Figure 7 shows a comparison of the relative chlorophyll content among varieties. In order to show the changes in pigment content in more detail, the changes in total chlorophyll, chlorophyll *a*, chlorophyll *b*, and carotenoid contents are shown. The pigment contents of the *Pink* and *Purple* varieties were high, which help improve the photosynthetic rate. Under NaCl treatment, net photosynthetic rate is obviously inhibited in all varieties (Fig. 8c). *Pink* shows the highest under saline condition, and the reduction rate under salt treatment is also the lowest in *Pink* (Fig. 8d). These results further explain why *Pink* has the highest biomass under NaCl treatment, which may be due to high accumulation in osmoregulation substance and high photosynthetic efficiency.

### Effects of NaCl on salt gland density of five varieties of Limonium sinuatum

Salt glands are structures for salt secretion that are specifically produced by recretohalophytes^47^. We therefore performed statistical analysis of the salt gland densities of expanded leaves of the five *L. sinuatum* varieties under NaCl treatment.

**Figure 9:**
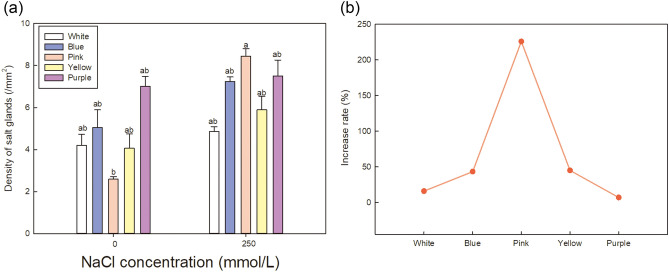
Effect of NaCl stress on salt gland density in the leaves of five varieties of *Limonium sinuatum*. (**a**) The data are means ± SD of five replicates. Different letters indicate significant differences between two groups at *P* = 0.05 using Duncan’s with SPSS. (**b**) The increase rate of salt gland density was calculated using (value under NaCl treatment—value under control)/value under control × 100%.

Figure 9a shows the changes in salt gland density in the leaves of the five varieties of *L. sinuatum* under salt stress (images shown in Supplementary Fig. 3). Salt gland density increased in all varieties under NaCl treatment compared to the control. The density of salt glands in *Pink* increased by 225.86% (Fig. 9b), while in *Purple* only 7%. The increased salt gland can help the plants to excrete more Na^+^ outsides in order to further decrease the Na^+^ accumulation in vivo.

**Figure 10:**
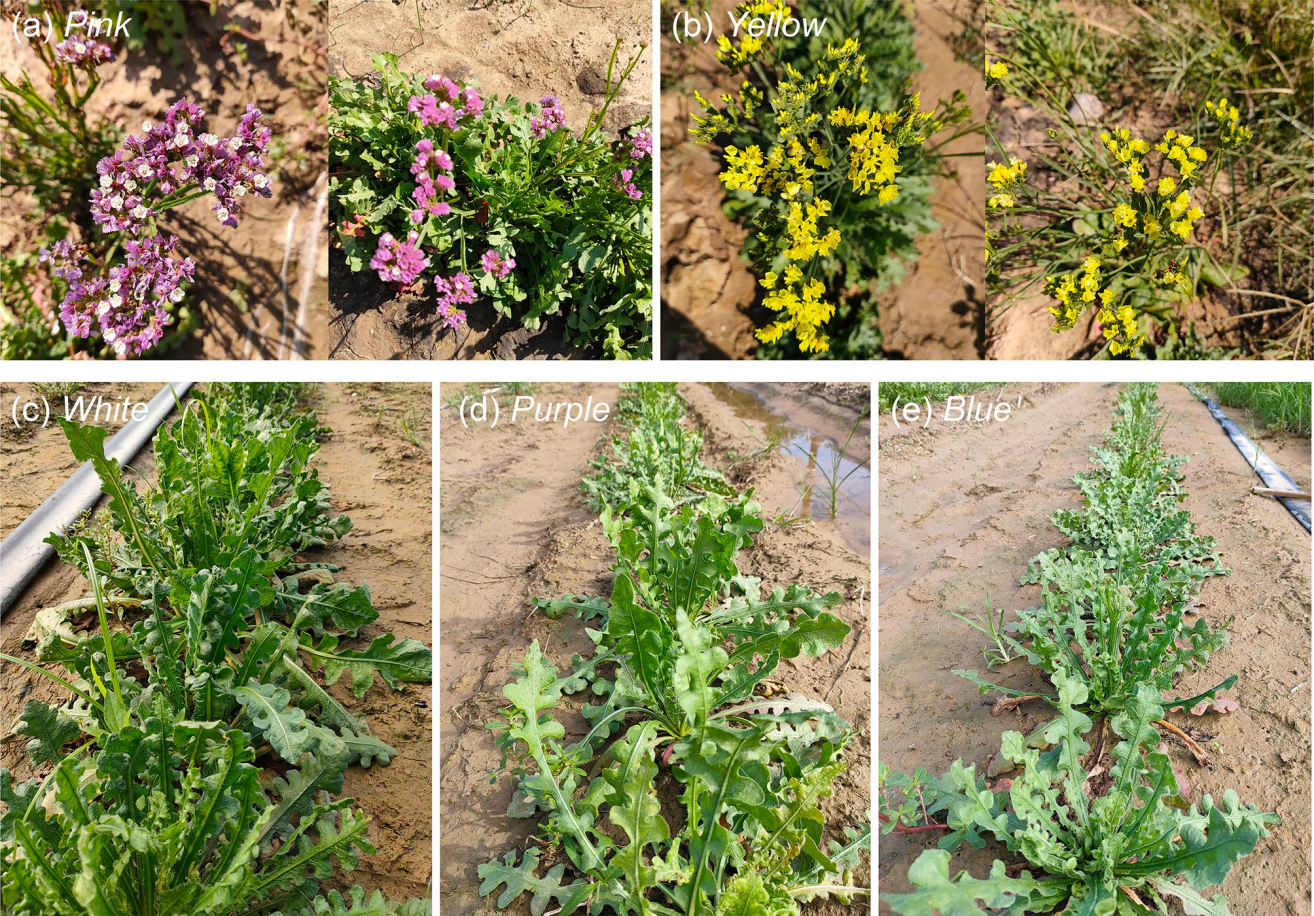
Growth of five varieties of *Limonium sinuatum* in saline soil.

Finally, given that *Pink* showed the greatest tolerance to NaCl treatment, in order to verify the optimal variety suitable for growing in field, we examined flowering in the five varieties grown in Yellow River Delta (salt content: 0.2%). After six months of growth, only *Pink* and *Yellow* plants flowered consistently, whereas the three other varieties rarely flowered and only showed vegetative growth (Fig. 10). These results suggest that *Pink* and *Yellow* are the optimal varieties for the development of saline horticulture and further planting in saline soil, which is consistence with the formal results in laboratory conditions.

## Discussion

*L. sinuatum* is a pioneer plant that could be used for the improvement of saline lands due to the high salt resistance and various colors^48^. Therefore, it is important to identify the best salt-tolerant varieties for cultivation in these areas. In the current study, *Pink* showed the highest biomass and the strongest salt resistance among the five varieties examined. Our analysis of physiological indicators including Na^+^, K^+^, Ca^2+^, and Cl^−^ contents; MDA, soluble sugars, proline contents, H_2_O_2_ content, relative water content and chlorophyll contents, osmotic potential, photosynthetic rate, and salt gland density under 250 mmol/L NaCl (salt-tolerance threshold) treatment explained why *Pink* has the greatest salt tolerance. Moreover, analysis of plants grown in the field (Fig. 10) confirmed the superior salt resistance of *Pink*. *Pink* is recommended as the optimal varieties for extensive planting and greening in saline soil, followed by *Yellow*.

Plant biomass is an important indicator of salt tolerance^43^: the greater the increase in biomass under salt stress, the higher the salt tolerance. As shown in Fig. 4, *Pink* showed the minimal reduction under 250 mmol/L NaCl treatment, and photosynthetic efficiency of *Pink* was also the highest among five varieties (Fig. 8), indicating that *Pink* suffered the least damage under saline condition*.* The three basic components of salt stress are usually considered as ionic toxicity, osmotic stress and oxidative stress^49^.

How *Pink* cope with high ionic toxicity? On the one hand, under salt stress, Na^+^ content increases, which affects the absorption of K^+^ and Ca^2+^. K^+^ plays an important role in the osmotic regulation of cells^50^. Ca^2+^ regulates the ionic balance and reduces the absorption of Na^+^^51^. In salt-tolerant plants, K^+^ efflux is significantly inhibited under salt stress to maintain high intracellular K^+^/Na^+^ levels, thereby reducing the damage from salt stress^52^. After salt treatment, the *Pink* variety had the relatively low Na^+^ and Cl^−^ contents among the five varieties, whereas K^+^ and Ca^2+^ showed the opposite trend. Therefore, *Pink* regulates ionic balance under salt stress, maintaining high K^+^/Na^+^ levels, thus showing strong salt tolerance. On the other hand, salt gland is the typical and specific epidermal structure of recetohalophytes^53^, which can excrete the excessive Na^+^ out of the plants to avoid damage^54^. The most salt gland was induced in *Pink* under salt treatment (Fig. 9), so it is speculated that Na^+^ can be effectively transferred out of the cell to further avoid ionic toxicity.

NaCl can induce the physiological drought due to the osmotic stress. Compatible media was always generated to deal with the osmotic stress, such as proline and soluble sugars. Proline is an important compatible solute in plant cells that protects enzymes from inactivation by NaCl and reduces the osmotic potential in the cells^55^, thereby helping plants resist external osmotic stress and tolerate adverse environments^29^. Sugar content under stress condition is intricately associated with carbohydrate content of plant^25^. Though not always the highest accumulation in proline and soluble sugars, *Pink* keep the relatively high level of osmotic adjustment substance to reduce the osmotic potential, which allowed the plant to re-absorb water from the NaCl solution to maintain normal growth.

Oxidative stress is inevitable induced by salt stress due to the generation of superoxide, with the typical representative H_2_O_2_^56^. *Pink* suffered the smallest oxidative stress under salt treatment and is more suitable for saline environment. MDA content can reflect the degree of membrane damage and the effects of stress on plants^57^. The least accumulation of MDA also insist the opinion that *Pink* suffered less oxidative stress.

Given that 16 physiological indicators were measured under salt treatment in addition to DW and FW, which one is most closely related to biomass? Correlation analysis was performed between FW and the other 14 indicators. As shown in Table 1, photosynthetic rate showed the strongest positive correlation with salt resistance in *L. sinuatum*.

**Table 1:** Correlation analysis between fresh weight (FW) and other 14 indicators including dry weight (DW), Na^+^ (Na), K^+^ (K), Ca^2^^+^ (Ca), Cl^−^ (Cl), MDA, soluble sugars (Sugar), proline (Proline), hydrogen peroxide (H_2_O_2_), relative water content (RWC) and chlorophyll contents (Chl), osmotic potential (Os), photosynthetic rate (Pr), and salt gland density (SG) using Pearson correlation analysis.

	Mean	Std. D	FW	DW	Na	K	Ca	Cl	MDA	Sugar	Proline	H_2_O_2_	RWC	Chl	Os	Pr	SG
FW	4.0040	1.55376	1.000														
DW	0.3427	0.14626	0.955**	1.000													
Na	0.4891	0.14593	− 0.030	0.011	1.000												
K	0.3658	0.10977	0.444	0.386	0.579*	1.000											
Ca	0.3192	0.05828	− 0.096	0.029	− 0.327	− 0.390	1.000										
Cl	0.0116	0.00171	0.120	0.207	− 0.209	− 0.303	0.452	1.000									
MDA	0.0202	0.00521	− 0.246	− 0.292	0.587*	0.330	0.046	− 0.046	1.000								
Sugar	0.8329	0.15517	0.349	0.365	0.193	0.006	− 0.265	− 0.226	− 0.012	1.000							
Proline	13.9340	4.86710	− 0.584*	− 0.445	− 0.431	− 0.703**	0.610*	0.315	0.038	− 0.273	1.000						
H_2_O_2_	22.8932	11.46676	0.003	− 0.004	0.654**	0.791**	− 0.437	− 0.497	0.418	0.059	− 0.384	1.000					
RWC	0.9145	0.01192	− 0.029	− 0.312	− 0.168	0.020	− 0.282	− 0.134	0.316	− 0.160	− 0.209	− 0.104	1.000				
Chl	0.6344	0.14499	0.316	0.431	0.069	− 0.001	− 0.086	0.126	− 0.645**	0.200	− 0.230	− 0.031	− 0.547*	1.000			
Os	− 2.7737	0.34859	− 0.488	− 0.402	0.119	− 0.051	− 0.123	− 0.269	0.201	− 0.041	0.406	0.392	− 0.180	− 0.224	1.000		
Pr	7.3313	1.09242	0.674**	0.591*	− 0.092	0.447	− 0.223	− 0.101	− 0.332	0.243	− 0.644**	0.049	0.023	0.179	− 0.677**	1.000	
SG	6.7918	2.46219	− 0.094	− 0.084	− 0.169	− 0.304	0.393	0.224	− 0.330	− 0.456	− 0.004	− 0.496	− 0.059	0.162	− 0.570*	0.147	1.000

**Correlation is significant at the 0.01 level (2-tailed).

*Correlation is significant at the 0.05 level (2-tailed).

Together, the results of our experiments show that the *Pink* variety has the strongest salt tolerance. Based on these results, the level of salt tolerance of the five varieties of *L. sinuatum* from high to low is *Pink*, *Yellow*, *Purple*, *White*, and *Blue*. Further analysis of the performance of these varieties in saline soil also validate the results of these preliminary experiments. Nonetheless, our findings provide a theoretical basis for the cultivation of *L. sinuatum* in saline-alkali areas, which could be widely planted to facilitate the greening and transformation of saline soils.

## Supplementary Information

Supplementary Information.
