# Supplementary material for: Salt-tolerance screening in Limonium sinuatum varieties with different flower colors
Source: Sci Rep. 2021 Jul 15;11:14562. doi: 10.1038/s41598-021-93974-3 (PMC8282669; doi:10.1038/s41598-021-93974-3)

**Salt-Tolerance Screening in *Limonium sinuatum* Varieties with Different Flower Colors**

Xiaojing Xu, Yingli Zhou, Ping Mi, Baoshan Wang*, Fang Yuan*

Shandong Provincial Key Laboratory of Plant Stress, College of Life Sciences, Shandong Normal University, Ji’nan, Shandong, P.R. China

*Corresponding author

**E-mail:**

Baoshan Wang: bswang@sdnu.edu.cn

Fang Yuan: yuanfang@sdnu.edu.cn

Tel.: 0086 531 86180197; Fax: 0086 531 86180197

**Supplementary figure legends**

**Supplementary Figure 1** Determination of the salt-tolerance thresholds of five varieties of *Limonium sinuatum*. A 50% reduction in biomass *vs.* the control was used as the standard to determine the salt-tolerance threshold.

**Supplementary Figure 2** Effect of NaCl stress on the fresh weight and dry weight of the leaves of five varieties of *Limonium sinuatum*. The data are means ± SD of three replicates. Different letters indicate significant differences between two groups at *P* = 0.05 using Duncan’s test with SPSS.

**Supplementary Figure 3** Effect of NaCl stress on salt glands in the leaves of five varieties of *Limonium sinuatum*.

Supplementary Figure 1


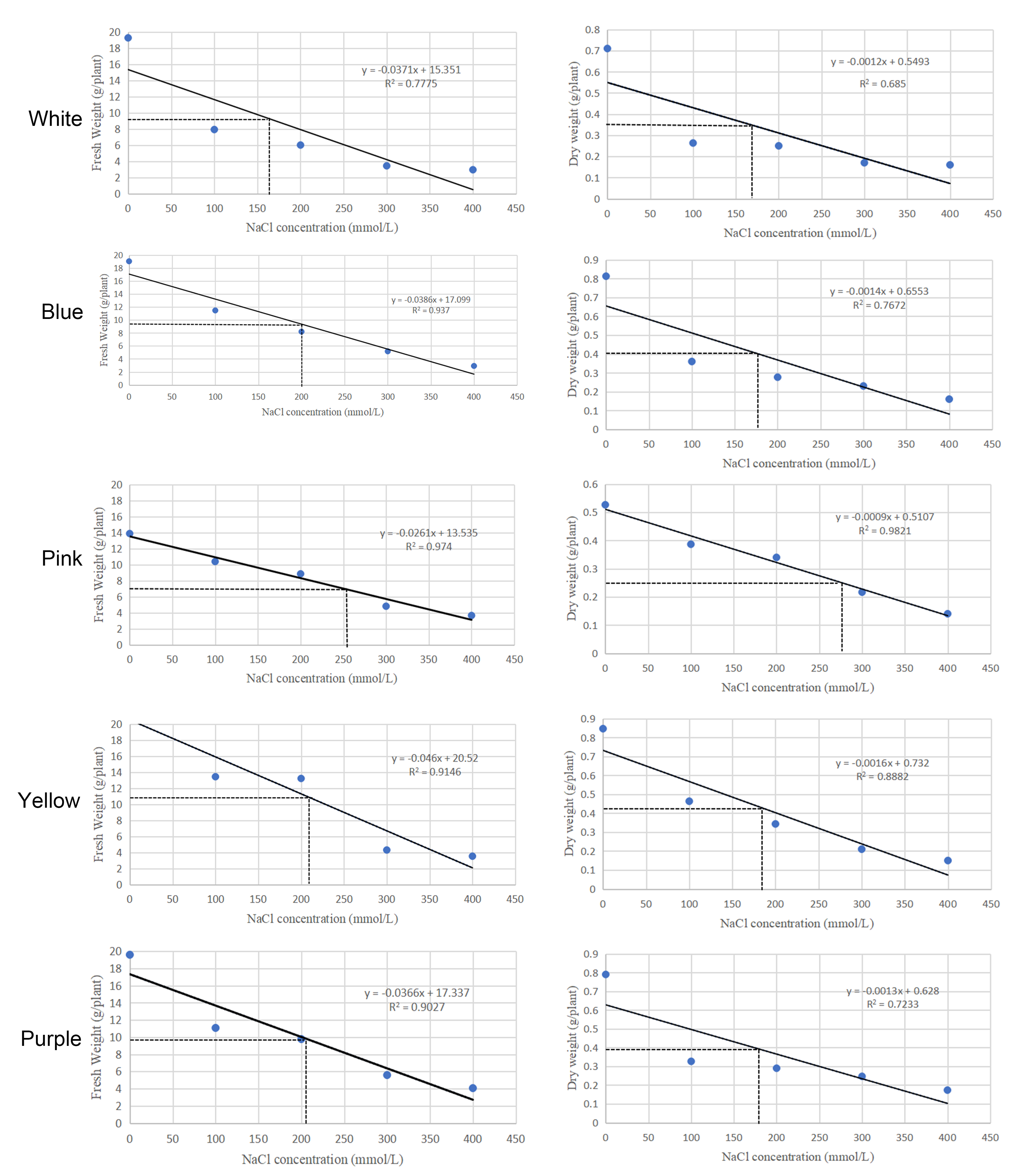


Supplementary Figure 2


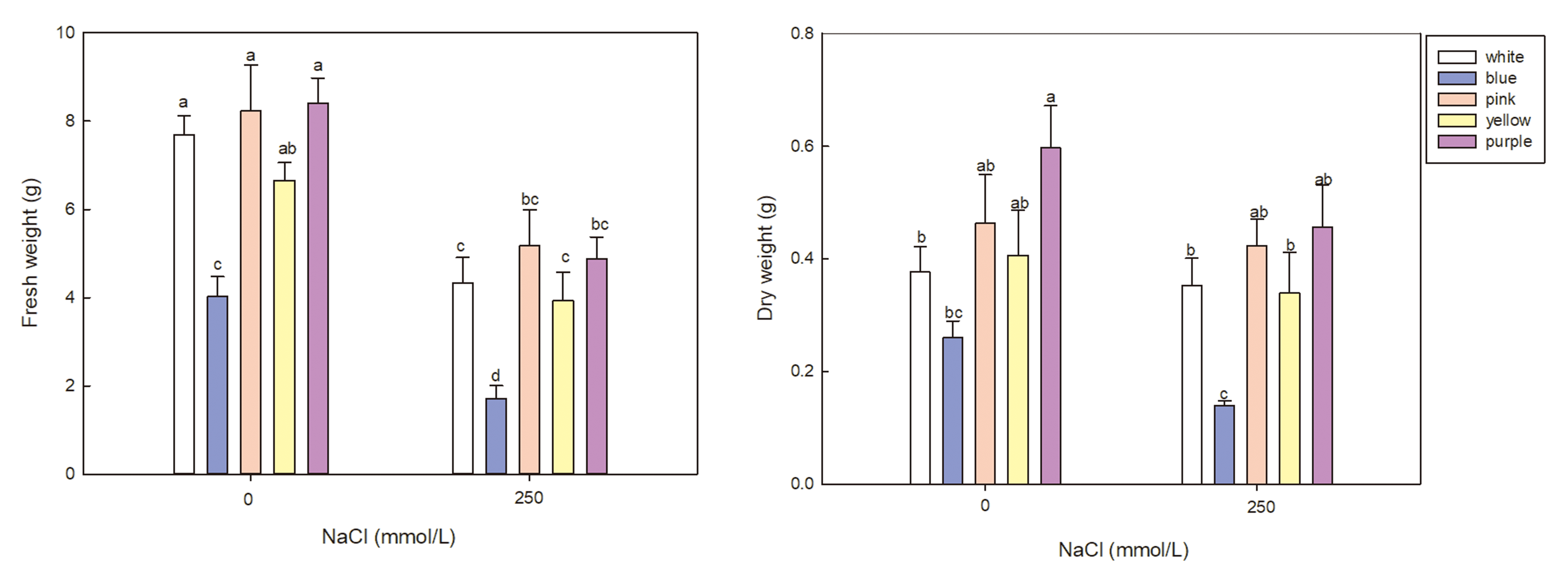


Supplementary Figure 3


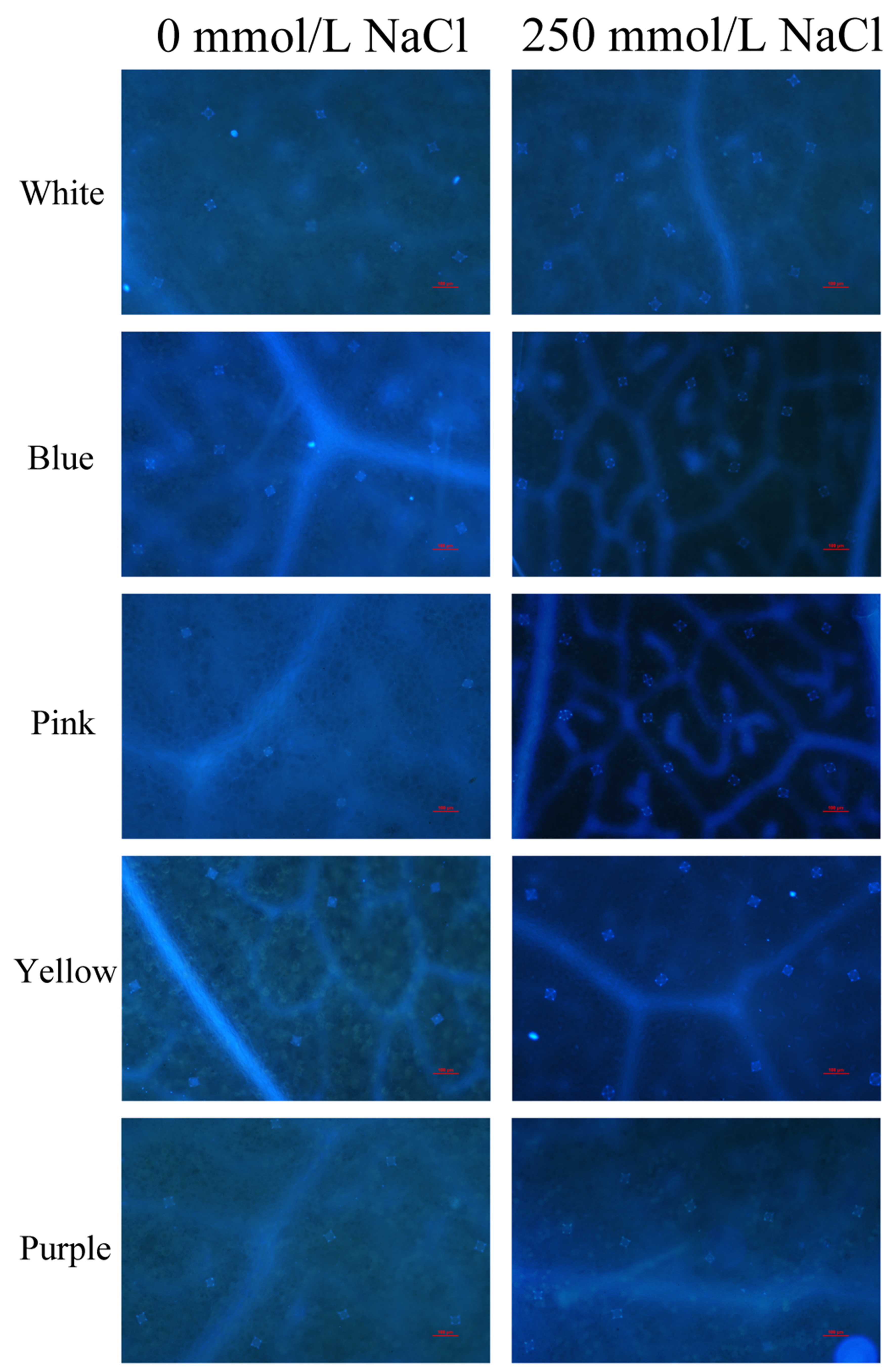

Supplement: Supplementary file 1 — Supplementary Information. [file 41598_2021_93974_MOESM1_ESM.docx]
